# Enhancing proficiency in ascites puncture through procedural simulation: an audit type study investigating medium-term skill retention

**DOI:** 10.1186/s12909-024-05063-4

**Published:** 2024-02-01

**Authors:** Aya Hammami, Hela Ghali, Nour Elleuch, Omar Khalil Ben Saad, Hanen Jaziri, Mehdi Ksiaa, Habiba Sik Ali

**Affiliations:** 1 Gastroenterology Department, Sahloul University Hospital, Sousse, Tunisia; 2 Department of prevention and security of care, Sahloul University Hospital, Sousse, Tunisia; 3 Faculty of Medicine of Sousse, University of Sousse, Sousse, Tunisia; 4 Medical intensive care unit, CHU Tahar Sfar, “LR12SP15”, Mahdia, Tunisia; 5 Faculty of Medicine, University of Monastir, Monastir, Tunisia

**Keywords:** Simulation, Pedagogy, Satisfaction, Evaluation, Learning

## Abstract

**Introduction:**

Medical simulation has become an essential teaching method for all health professionals. It not only allows to acquire technical and non-technical knowledge, but also helps the maintenance of acquired knowledge in the medium and long term. Ascites puncture is part of the basic technical procedures learned by medical students during their internship.

**Objectives:**

To evaluate the role of simulation-based learning of ascites puncture on the improvement of theoretical knowledge and maintenance of skills at 3 months.

**Methods:**

We conducted an audit type study with two cycles of data collection at the simulation center at the Faculty of Medicine of Sousse between November 2020 and June 2021. We included learners in their third year of medical studies who had a hospital internship in the gastroenterology department at Sahloul Hospital in Sousse. All learners attended the initial simulation session on ascites fluid puncture. Thereafter, they were free to accept or refuse participation in the evaluation session that was scheduled after 3 months, depending on their availability.

**Results:**

Forty learners participated in the procedural simulation of the ascites fluid puncture technique. Thirty-four (85%) were female and six (5%) were male. In our study, we showed that following procedural simulation training of ascites puncture, there was a significant improvement in the theoretical knowledge of the learners (*p* < 0.000). Objective assessment of technical skills after 3 months showed the benefit of performance maintenance (*p* < 0.000).

**Conclusion:**

Our study confirmed the benefit of simulation-based learning on the improvement of theoretical knowledge and the maintenance of technical performance in the medium term.

## Introduction

Ascites puncture is a procedure common to many specialties, including hepatology, gastroenterology, and oncology. It is one of the basic technical procedures learned by medical students, although no formal training is provided.

For an extended period, practitioners acquired this procedure by observing and learning at the patient’s bedside under the guidance of an intern, resident, or senior doctor, and the first puncture was usually performed on a patient. Learned societies have not issued specific recommendations regarding the training or skills necessary to perform an ascites puncture.

The rate of immediate complications from ascites puncture is relatively low, making the procedure safe when performed according to current recommendations, and often compatible with outpatient management [1, 2]. Minor complications, such as wall hematomas, occur in 1% of cases, and severe complications, such as hemoperitoneum or bowel perforation, occur in less than one case in 1000 [3].

However, the diagnostic and therapeutic importance of this procedure, and the negative experience for the patient, due to the pain and stress that could be caused by an ascites puncture performed by a medical trainee or a young doctor with little or no training, argue in favor of structured training in this procedure.

Since 2019, the Faculty of Medicine in Sousse has implemented procedural simulation teaching for ascites puncture specifically designed for learners undergoing placements in the gastroenterology department at the Sahloul University Hospital in order to make up for the shortcomings of learning in a clinical environment.

Simulation, which has become an essential teaching tool, not only enables technical and non- technical knowledge to be acquired more quickly and without risk to the patient, but also helps to maintain the knowledge acquired over the medium and long term [4]. It is then necessary to address the lacunae in formal training for ascites puncture.

This study aimed to evaluate retention in learning ascites puncture by simulation at 3 months, as well as learners’ satisfaction with the simulation session itself.

## Methods

### Type and location of study

This is an audit type study with two cycles of data conducted at the simulation Centre at the Faculty of Medicine in Sousse (CESIME) between November 2020 and June 2021.

### Study population

#### Inclusion criteria

We included students in their third year of medical studies (DCEM1), enrolled at the Faculty of Medicine in Sousse, who had completed a hospital placement in the gastroenterology department at the Sahloul Hospital in Sousse.

#### Exclusion criteria

We excluded students in their third year of medical studies (DCEM1), enrolled at the Faculty of Medicine in Sousse, who had not completed a hospital placement in the gastroenterology department at the Sahloul Hospital in Sousse.

The hospital placement lasted ten half-days.

The number of learners varied between 12 and 15 per group. All learners attended the initial simulation session on puncturing ascites fluid. Thereafter, they were free to accept or refuse participation in the evaluation session, which was scheduled after 3 months, depending on their availability.

### Procedure

A simulation session on exploratory puncture of ascites fluid was scheduled for Monday morning of the 2nd week of the course. The simulation sessions occurred at the Simulation Centre of the Faculty of Medicine in Sousse, within a room meticulously designed to replicate the environment of a gastroenterology hospital room, with all the necessary equipment. The total duration of the simulation session was 90 min. The session was preceded by theoretical training.

The first phase of the study was to provide theoretical training for all the learners, by giving them a self-learning module and a video explaining the procedure on the Tunis Virtual University (UVT) platform. Learners were invited to consult these documents a week before the simulation session, and were divided into small groups of six.

After welcoming the learners, the training always began with a pre-test, consisting of five multiple-choice questions (MCQs).

This stage was used to assess the learners’ knowledge and prerequisites in order to determine their needs.

The next stage was the training itself. The course of the procedural simulation session followed the classic model, with an initial pre-briefing phase, the practical situation, which took place at the same time as the analytical debriefing, followed by a summary debriefing.

The briefing lasted 10 min. Its purpose was to present the educational objectives of the training session and to allow the learners to familiarize themselves with the dummy, its environment, and the equipment available.

We used a multitasking dummy with an orifice (stoma) which was used for the puncture point. We were also reminded of the rules of confidentiality, based on an oral agreement. Supervision was provided by the same trainer.

No video recording was made.

The simulation and analytical debriefing lasted around 60 min. For the simulation session itself, we adopted the “four-stage learning” approach, organized as follows:

- First stage: the trainer demonstrates the ascites fluid puncture sequence in real-time without comment.
- Second stage: the trainer demonstrates how to carry out the sequence, describing each step.
- Third stage: the learners explain the techniques while the trainer carries them out.
- Fourth stage: each candidate takes it in turns to complete the sequence, while receiving feedback in real time.

The analytical debriefing was given in real time.

The simulation session was followed by a 20-minute summary debriefing, led by the training doctor. The aim was to encourage the participants to give constructive feedback in line with good debriefing practice. At the end of the training session, the trainer reviewed the points learned and gave feedback to the learners on the session as a whole.

At the end of the course, all the trainees were assessed by a post-test comprising the same MCQs presented in the pre-test, and a grid detailing the different steps involved in performing ascites puncture in order to assess their acquisition of this technical skill. The grid comprised ten steps. The trainer had to tick “done” or “not done” on the grid and then calculate the total score by awarding one point for each of the steps performed by the learner.

Each session ended by thanking the learners for their participation. They were given a certificate of attendance, as well as consolidation and progression documents. Learners’ perceptions of the simulation-based learning sessions were assessed using an anonymous questionnaire distributed at the end of the session, and according to a qualitative Likert-type scale [5] (typical responses: 1: Strongly disagree, 2: Somewhat agree, 3: Somewhat disagree, 4: Strongly agree).

3-month skills retention assessment.

All the trainees who took part in the study were invited to attend a separate meeting 3 months after the initial practical training, during which none have had other training sessions. Each learner was invited to perform an ascites puncture on the same manikin and was assessed using the same grid devised during the initial training. A second score was calculated for each learner.

### Statistical analysis

The data collected were entered and analyzed using SPSS Statistics 21 software.

The distribution of quantitative variables was verified using the Kolmogorov-Smirnov test.

Variables with a normal distribution were expressed by their means ± standard deviation, and those with a non-normal distribution by their median and interquartile range (IQR) [25th percentile– 75th percentile].

The mean scores for the pre-test and post-test were compared using Student’s paired-sample t-test.

The medians of the scores for the first and second technical assessments were compared using the Wilcoxon test.

Qualitative variables were expressed as numbers and percentages.

The p level of statistical significance was set at 0.05.

### Ethical consideration

This research was approved by the ethics committee of The Sahloul University Hospital number HS 08-2020. All students consented to the participation in this study.

## Results

We included 40 students in the second cycle of medical studies during their stay in the gastroenterology department at the Sahloul University Hospital during the study period. There were 34 girls (85%) and 6 boys (15%) (Sex ratio F/M: 5.6), with a median age of 21.53 years [IQR: 21–23 years]. None of the learners had previously attended a simulation session in another discipline.

We found a statistically significant improvement in the marks obtained after the training (2.93.

**Fig. 1:**
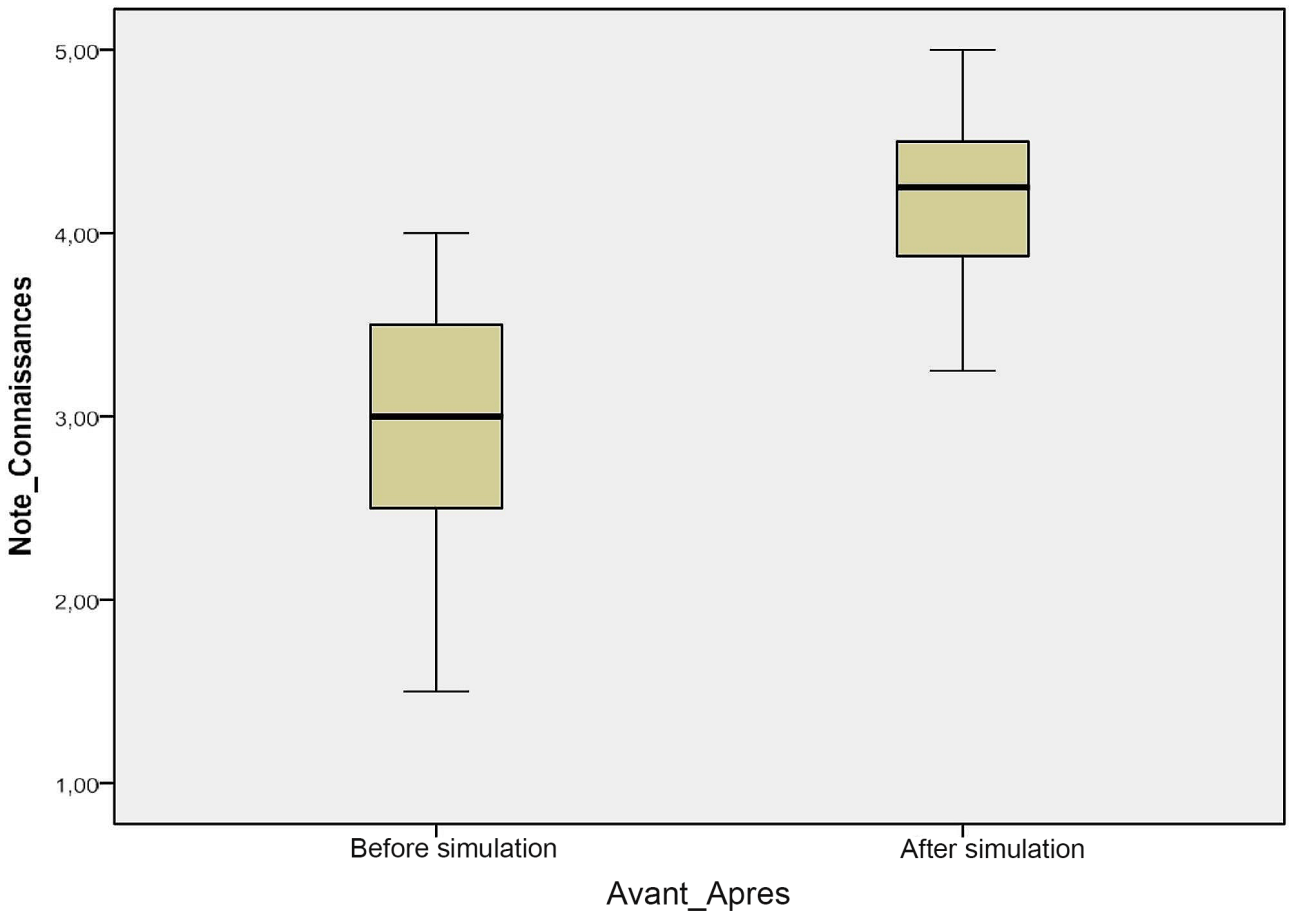
Comparison of pre-test and post-test averages

± 0.71 out of 5 versus 4.21 ± 0.94) (*p* < 0.000) (Fig. 1). In our study, all learners (100%) were able to improve their knowledge following the proposed training.

After 3 months of the initial simulation session, we noted a statistically significant improvement in technical skills, with statistically significant mastery of 2 items: preparing the equipment required for puncture (*p* < 0.001) and wearing sterile gloves (*p* < 0.001) (Table 1).

**Table 1:** Results of the assessment of technical skills at the initial simulation session and after 3 months

Deed done	Initial simulation N (%)	Simulation at 3 months N (%)	Value of p
Preparing your equipment	25 (62,5)	37 (92,5)	< 0,001
Wear sterile gloves	15 (37,5)	35 (87,5)	< 0,001
Note the puncture site: at full maturation, at the junction of the outer and middle thirds of the line joining the left anterior superior iliac spine and the umbilicus.	22 (55)	29 (72,5)	**0,10**
Disinfect the skin with betadine	36 (90)	39 (97,5)	**0,35**
Place a sterile field with holes in it, centered on the area where you are going to inject	21 (52,5)	22 (55)	**0,82**
Prick the puncture site perpendicular to the abdominal wall	40 (100)	40 (100)	1,00
Take a sample of ascites using a 50 ml syringe	40 (100)	40 (100)	1,00
Re-disinfect the puncture site	33 (82,5)	29 (72,5)	**0,28**
Apply a sterile compress and plaster to the puncture site	18 (45)	22 (55)	**0,37**
Dispenseascitesfluidintovialsfor biochemical and bacteriological examination	**40 (100)**	**40 (100)**	1,00

*Note*: The results in bold reflect the number and percentage of participants who “totally agree” with the item

**Fig. 2:**
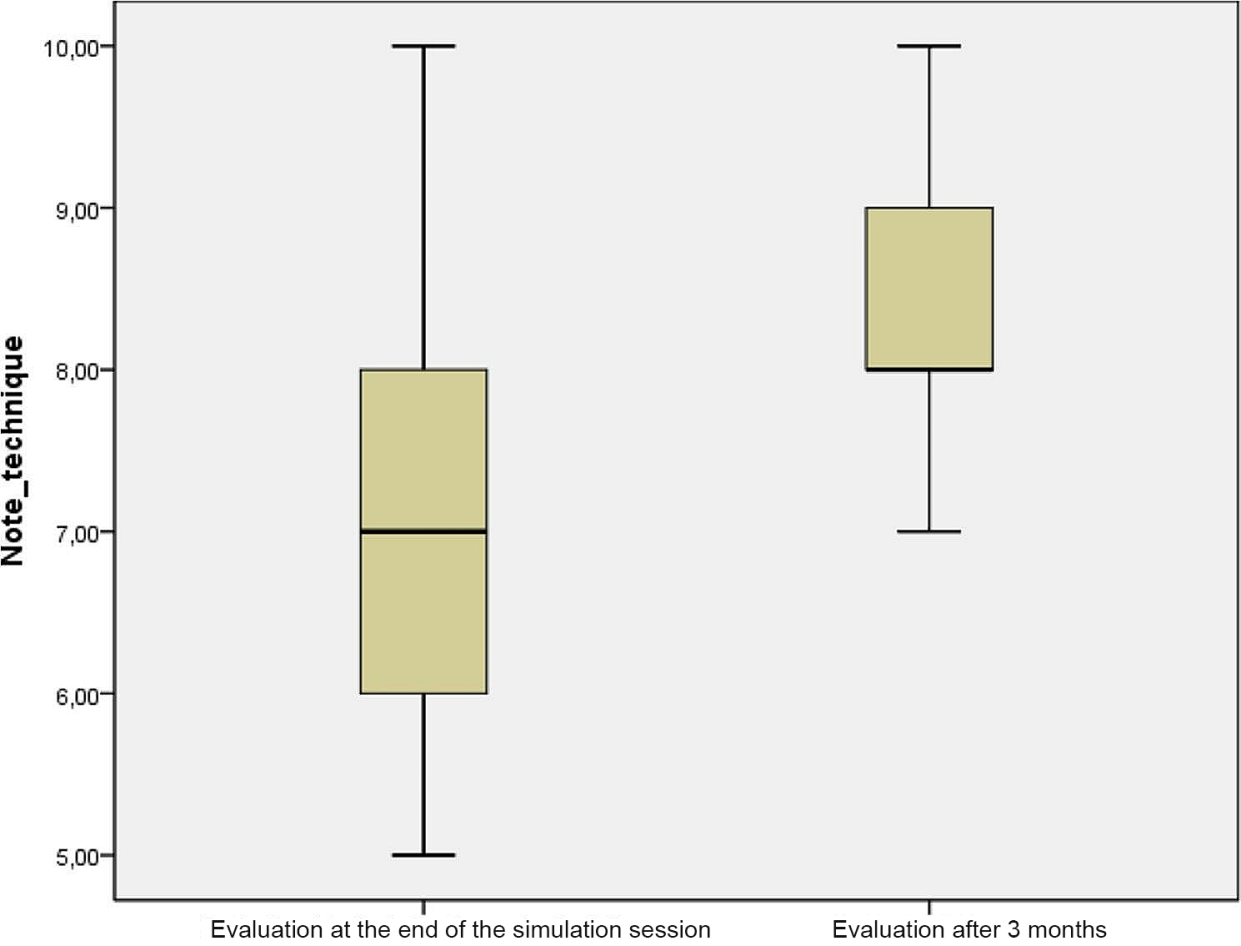
Assessment scores obtained during the initial simulation and at 3 months

The median score for the initial assessment of technical skills at the end of the simulation session was 7 [6–8] /10 versus 8 [8–9] /10 at the 3-month assessment, with a statistically significant difference (*p* < 0.001) (Fig. 2).

More than two-thirds felt that the duration of the session was appropriate for achieving the objectives, and 85% were very satisfied with the general atmosphere of the training. In terms of scientific interest, the majority of learners (80%) felt that the theme of the course was relevant, and more than half felt that the course complemented the practical course (Table 2). More than half the students (65.5%) felt that the briefing was reassuring and that it reduced the stress experienced during the procedure. The allocation of speaking time was considered fair by 75% of participants. According to 67.5% of learners, simulation training enabled them to make progress in their thinking (Table 2).

**Table 2:** Assessment of learner satisfaction with the simulation session on ascites puncture

Item	I totally disagree N (%)	Somewhat disagree N (%)	Somewhat agree N (%)	I totally agree N (%)
Satisfaction with the organization				
The length of the session is adapted to the content	0	1 (2,5)	10 (25)	**29 (72.5)**
The allocation of time between the different phases of the simulation session was appropriate	0	2 (5)	10 (25)	**28 (70)**
The size of the group was appropriate	2 (5)	6 (15)	14 (35)	**18 (45)**
The general atmosphere was conducive to learning	0	1 (2,5)	5 (12,5)	**34 (85)**
Satisfaction with scientific interest				
The theme of the session is relevant	0	1 (2,5)	7 (17,5)	**32 (80)**
The session helped me to make links with my previously acquired theoretical knowledge	0	1 (2,5)	11 (27,5)	**28 (70)**
The session complements the practical work done on the course	0	3 (7,5)	13 (32,5)	**24 (60)**
Satisfaction with educational value				
The session was interactive	0	1 (2,5)	5 (12,5)	**34 (85)**
The situation proposed was close to reality	0	4 (10)	16 (40)	**20 (50)**
The documents provided are relevant	1 (2,5)	6 (15)	16 (40)	**17 (42,5)**
Satisfaction with the way the session was run				
The trainers gave participants a warm welcome	0	0	9 (22,5	**31 (77,5)**
The briefing reassured me	0	1 (2,5)	13 (32,5	**26 (65,5)**
During the briefing, all these elements were discussed: the value of the simulation, ethical principles, the objectives of the session, the distribution of each person’s roles, etc.	0	1 (2,5)	12 (30)	**27 (67,5)**
During the debriefing, the players were able to express their feelings	0	1 (2,5)	8 (20)	**31 (77,5)**
Everyone was given a fair share of speaking time	1 (2,5)	2 (5)	7 (17,5)	**30 (75)**
There was no judgment at the debriefing	0	3 (7,5)	6 (15)	**31 (77,5)**
I had the opportunity to reflect on and discuss my performance during the debriefing (for those who took part in the role-play).	0	0	8 (20)	**32 (80)**
Where necessary, the trainer offered me clues to help me make progress in my thinking.	0	1 (2,5)	12 (30)	**27 (67,5)**
The trainer provided constructive criticism during the debriefing.	0	0	7 (17,5)	**33 (82,5)**
The trainer summarised the key issues during the Debriefing	0	**1 (2,5)**	**12 (30)**	**27 (67,5)**

*Note*: The results in bold reflect the number and percentage of participants who “totally agree” with the item

The overall quality of the simulation session was very satisfactory according to the majority of learners (82.5%).

## Discussion

In our study, we showed that following procedural simulation training in ascites puncture there was a significant improvement in the theoretical knowledge of DCEM1 learners, as evidenced by the significant improvement in the mean score of the pre-test from 2.93 ± 0.71 out of 5 to 4.21 ± 0.94 in the post-test (*p* < 0.000).

Objective assessment of technical skills after 3 months of the simulation session showed the benefit of a simulation session in maintaining performance (*p* < 0.000).

The learners’ perceptions were confirmed by a satisfaction survey at the end of the session, which showed that the majority of learners (82.5%) were satisfied overall with simulation- based learning.

In many countries, the learning of medicine has been enriched by the addition of simulation, an innovative teaching method based on real-life situations and enabling learning in a safe environment, both for the learner and the patient [6].

The value of using this learning method has been highlighted, particularly since the report “To err is human” published in 1990 by the Institute of Medicine in the United States. This report revealed the high prevalence of deaths due to human factors, estimated at over 98,000 per year [7].

Experiential learning does not replace other learning methods (abstraction, observation or companionship) [8], but complements them by activating and putting knowledge into practice in a safe environment. Simulation tools are diverse and each tool is chosen according to the educational objectives being pursued [9, 10].

The problem with this method was that the learners performed the first procedures in a non-standardized way on real patients, exposing them to sometimes major risks such as hemoperitoneum, with a prolonged learning period.

The first study to evaluate the impact of teaching ascites puncture by simulation was published in 2011 by an American team [11].

In 58 first-year medical interns trained during a 3-hour teaching session combining theoretical reminders, a demonstration and training on an ultrasound-guided simulator, the training led to an improvement in performance on a 25-item score from 33% in the pre-test to 93% in the post-test (*p* < 0.05) [11].

A second, more recent study by Tejos et al. [12], included 247 learners during their gastroenterology clerkship who underwent simulation training in ascites puncture using a simulation model specifically designed and validated for abdominal paracentesis. Comparing the results of the pre- and post-tests, they noted that the learners had significantly improved their skills [13.36 ± 4.46 (55.7%) vs. 22.3 ± 1.83 (92.9%) respectively, *p* < 0.001].

Learners rated the training as very satisfactory with an average of 4.8 ± 0.38 on a Likert scale of 1 to 5.

Procedural simulation using low-fidelity mannequins is a tool for consolidating theoretical knowledge and acquiring technical skills. The type of dummy used and its level of realism and sophistication must therefore be based above all on the precise teaching objectives. The visual and morphological realism of the equipment, like the realism of the environment or the psychological realism, serve only one purpose: to immerse the participant in professional life [13].

To date, there is no standardized model for teaching ascites puncture.

A team of gastroenterologists and surgeons in Chile designed a specific, realistic simulator for ascites puncture [13]. The model, validated by a group of experts, consisted of a rigid case with a bag that simulated intra-abdominal pressure and a puncture zone with a 10 mm thick patch with different layers, which allowed easy traction of the skin.

The innovative features of this paracentesis model led to a patent application.

A second Brazilian team had invented a “low-cost” model for teaching ascites puncture. This model consisted of a plastic dummy in which a circular incision was made over the entire abdomen to simulate the abdominal cavity, water-filled gloves, gummed tape, sponges and synthetic leather. These materials were chosen because of their availability, low cost and reproducibility [14].

Unfortunately, the simulation Centre at the Faculty of Medicine in Sousse does not have a dummy specifically for ascites puncture. Instead, We utilized a multitasking mannequin equipped with a stoma orifice, to which we attached a bottle filled with water. The learners considered this orifice to be the puncture point, which lacked realism and fidelity.

A great deal of work has been done to evaluate the impact of simulation-based teaching on the quality of the teaching itself, and on the possible improvement in practices that it could engender. The evaluation of such training is classically carried out using Kirkpatrick’s classification, from which we drew our inspiration [15, 16]. This classification comprises 4 increasing levels of training, which may be summarized as follows: satisfaction, contribution of knowledge, change in practice and clinical outcome.

In our study, participants’ assessments were positive. Overall, all the participants were satisfied with the teaching and most said that they had benefited significantly from the session for their subsequent professional practice. Students’ perceptions of the quality of teaching through simulation were generally positive and supported the hypothesis that simulation contributes to safer practice [17, 18].

Most studies to date have focused only on immediate benefits and short-term skills retention, with the longest studies ranging from three to six months [19].

Some authors have studied the effect of high-fidelity simulation on knowledge retention in nursing learners, and the results were very divergent. Two studies, by Tawalbeh et al., and Ackermann et al., showed significant knowledge retention 3 months after high-fidelity simulation [20, 21]. In both studies, the learners in the experimental group had a significantly higher average score than the other learners in the control group.

The results of our work were consistent with those of the above-mentioned studies, confirming the benefit of simulation training in maintaining technical performance for 3 months. This improvement could be explained by the quality of the debriefing given during the initial simulation session. In fact, each learner benefited from an individual debriefing to highlight the positive points and the points to be improved in the practice of the procedure, and continued to have a refresher at each feedback given to the other participants, and during the collective debriefing [22].

Positive reinforcement in the form of a certificate of participation in the simulation session, and the creation of an environment conducive to teaching could also be factors that encourage skills retention.

However, the study by Tubaishat et al. [23], which evaluated the effect of simulation on the knowledge of two groups of nursing learners (experimental group and control group) using a clinical scenario on cardiac arrhythmias, did not confirm these data. By comparing the results of the pre-test and the first post-test, the authors had shown a significant difference between the two groups of learners. Learners in the experimental group showed a significantly greater gain in knowledge than those in the control group. However, 3 months after the simulation session, the results of the second post-test showed no significant difference between the groups, which meant that the effect of simulation on knowledge retention could not be retained [23].

Similarly, Stefanidis et al. [24] compared the contribution of simulation to skill maintenance after a simulation of laparoscopic suturing in two groups of learners (experimental vs. control).

The results of this study showed that the experimental group performed better than the control group at the immediate post-simulation assessment and after 5 months (*p* < 0.001). For the experimental group, a significant decline in performance was observed at 5 months in the absence of practice (*p* = 0.05).

The main limitations of our work are linked, on the one hand, to the absence of any evaluation of the long-term maintenance of performance, which does not allow us to predict the impact on the acquisition of gestures in a real situation, and, on the other hand, to the absence of any evaluation of the patient’s communication and information skills.

The study recommends the implementation of standardized simulation models specifically designed for ascites puncture to improve the training sessions, extending the evaluation period to assess long-term performance and the incorporation of assessments for patient communication and information skills to ensure a comprehensive evaluation of learners’ preparedness for real-world scenarios.

## Conclusion

The imperative for improved medical training finds resonance in the widespread adoption of medical simulation as a pivotal teaching method. Although the integration of simulation-based teaching into practical clerkships is feasible and well-received by trainees, its transformative potential is limited in impacting immediate clinical practice. Recognizing this, there is an urgent need to refine and implement simulation-based education strategies, especially in resource-constrained settings, to bridge the gap between theoretical knowledge and practical skills, ultimately advancing patient care in line with the foundational principle of “never the first time on the patient.”

## Data Availability

Data is available upon reasonable request from corresponding author.
